# Genomic landscape of pathogenic mutations in Pakistani population with late-stage colorectal cancer

**DOI:** 10.12669/pjms.41.5.10265

**Published:** 2025-05

**Authors:** Zeeshan Ansar, Asghar Nasir, Tariq Moatter, Uzma Shamsi

**Affiliations:** 1 Zeeshan Ansar Department of Pathology and Laboratory Medicine, Aga Khan University, Karachi 74800, Pakistan; 2 Asghar Nasir Department of Pathology and Laboratory Medicine, Aga Khan University, Karachi 74800, Pakistan; 3 Tariq Moatter Department of Pathology and Laboratory Medicine, Aga Khan University, Karachi 74800, Pakistan; 4 Uzma Shamsi Department of Community Health Sciences Aga Khan University, Karachi 74800, Pakistan

**Keywords:** Colorectal cancer (CRC), KRAS, Microsatellite instability (MSI), Next-generation sequencing (NGS), Pathogenic mutations, TP53

## Abstract

**Objective:**

To assess the frequencies of pathogenic mutations in Pakistani population with late-stage Colorectal cancer (CRC).

**Methods:**

This was a descriptive analysis of CRC patients who got their next-generation sequencing (NGS) tests (targeted panel) done at AKUH, Karachi between January 2021 and December 2021. Pathogenic variants were identified using American College of Medical Genetics and Genomics (ACMG) classification.

**Results:**

Among the 35 CRC patients analyzed, 31.4% were < 50 years old and 60% were males. Mutation analysis showed a high prevalence of TP53 mutations in 23 patients (65.7%). KRAS mutations were detected in 19 patients (54.3%) Other mutations included PIK3CA in 3(8.6%), NRAS in 3(8.6%), EGFR in 3(8.6%), and MET in 1(2.9%). Double gene mutation (KRAS and TP53) were observed in 13 (37.1%) and (PIK3CA and KRAS) in 2 (5.71%) samples. A triple gene mutations (KRAS, TP53, and PIK3CA) were found in 1 (3%) of CRC tumors. The remaining samples were wild type for genes analyzed. Microsatellite instability (MSI) status was assessed, revealing 2.9% MSI-high tumors, 37.1% MSI-stable tumors, and a concerningly high proportion (60.0%) of samples where MSI testing was not performed.

**Conclusion:**

This study highlights distinct a genetic profile of CRC in the Pakistani population, characterized by a significant prevalence of TP53 and KRAS mutations.

## INTRODUCTION

Colorectal cancer (CRC) is a heterogeneous disease characterized by diverse genetic alterations during its carcinogenesis. These alterations define distinct molecular subtypes and include somatic mutations in genes such as *APC, KRAS, BRAF, and TP53*. CRC also exhibits microsatellite instability (MSI) and chromosomal instability (CIN). While MSI status has prognostic and predictive value, particularly for immunotherapy response, *KRAS* mutation status is a critical and widely used molecular marker, especially in metastatic CRC, for predicting response to EGFR-targeted therapies. The frequency and specific mutations observed in CRC vary significantly depending on the tumor’s subtype, stage, and location. Mutations in the *KRAS* and NRAS genes occur in approximately 40% of metastatic CRC (mCRC) samples.^1^ Patients with metastatic CRC and *KRAS* mutations have the poorest prognosis.^2^,^3^ However, two NGS studies found no outcome difference between KRAS mutations and *KRAS* / *NRAS*/ *BRAF* genotype mutations.^4^,^5^ Studies consistently show *KRAS* wild-type CRC tumors are associated with better survival than mutant *KRAS* tumors.^6^-^9^

NGS provides comprehensive genetic profiling in CRC, including less common but clinically relevant genes for prognosis and therapy. Multigene NGS panels effectively identify CRC susceptibility genes.^6^ In Pakistan, CRC incidence is rising in younger populations, who often present with advanced-stage disease and poorer prognosis.^7^

To address the gap in genomic data for CRC in Pakistan, this study examined pathogenic mutations in late-stage (III & IV) CRC from Pakistani adults.

## METHODS

This is a descriptive study conducted in cases with CRC who underwent next-generation sequencing (NGS) tests at Aga Khan University hospital, Karachi Pakistan between January 2021 and December 2021.The study employed a purposive sampling strategy, selecting confirmed cases of CRC that had undergone next-generation sequencing. It included information on the patient’s age, gender, histopathology reports (grade, tumor site and TNM AJCC stage) and mutation data. The diagnosis of malignancy was confirmed based on morphology and histochemical features.

### Ethical Approval:

The study was approved by the ethical review committee of the Aga Khan University Hospital approved the project (ERC # 7741, dated November 22, 2021).

### Analysis of CRC tumor samples using NGS:

Formalin fixed, paraffin embedded (FFPE) tumor samples were assessed for sufficiency and tumor rich areas identified by a certified pathologist, the tumor area was marked on a hematoxylin-eosin slide by a histopathologist, and more than 30% of tumor content was confirmed before DNA extraction with a minimum tumor tissue surface 140 mm2 with at least ≥30% nucleated tumor cells required. DNA was extracted from macro dissected FFPE tissue. DNA extraction was performed using the QIAamp DNA FFPE Tissue Kit. The quality of extracted DNA was evaluated using an absorbance ratio of 260 nm to 280 nm (A260/A280) and 260 nm to 230 nm (A260/A230). The purity criterion for samples with the A260/A280 ratio is within the range of 1.8–2.0, and the A260/A230 ratio is within 2.0–2.2

Molecular analysis used 20 ng DNA with a commercially available NGS targeted panel (TruSight Tumor 15, TST15, Illumina) sequenced on the MiSeq platform (2 × 150 bp configuration, Illumina). The TST15 includes regions of 15 genes covering hotspot variants, including single nucleotide variants and small insertions/deletions in the *AKT1, BRAF, EGFR, ERBB2, FOXL2, GNA11, GNAQ, KIT, KRAS, MET, NRAS, PDGFRA, PIK3CA, RET* and *TP53* genes. Bioinformatic analysis used MiSeq Reporter with a manufacturer supplied TST analysis module (Illumina). The identification of genetic variants and their clinical significance was determined using the following resources:

### ClinVar:

A publicly accessible, free archive of reports of the relationships among human variations and phenotypes, with supporting evidence. (https://www.ncbi.nlm.nih.gov/clinvar/)

COSMIC (Catalogue of Somatic Mutations In Cancer): A comprehensive resource for exploring somatic mutations in human cancer. (https://cancer.sanger.ac.uk/cosmic)

### Ensembl:

A genome browser for vertebrate genomes that supports research in comparative genomics, evolution, variation and regulatory genomics. (https://asia.ensembl.org/info/index.html)

Identification of somatic effects were reported based on American College of Medical Genetics (ACMG) guidelines. Patients’ demographic, histopathological, and mutation data were recorded in a Microsoft Excel sheet.

## RESULTS

Thirty five samples were collected in accordance with the sample preparation procedures mentioned above. Table-I shows that patients were predominantly male, 21 (60%), and over 50 years of age, 24 (68.6%). Among the 35 samples, stage information was available for 21 samples. Of those with available staging data, two samples (9.5%) were stage-3, and 19 samples (90.5%) were Stage-4. The distribution of tumor sites was: five samples (21.7%) in the proximal colon, 14 samples (60.9%) in the distal colon, and four samples (17.4%) in the rectum. Most tumors were moderately differentiated, with 14 samples (60.9%). Mutation analysis revealed *TP53* mutations in 23 samples (65.7%) and *KRAS* mutations in 19 samples (54.3%). Other mutations included *PIK3CA* in three samples (8.6%), NRAS in three samples (8.6%), EGFR in three samples (8.6%), and MET in one sample (2.9%). No mutations were detected in KIT, *BRAF*, or ERBB2. For microsatellite instability (MSI) status, one sample (2.9%) was classified as MSI-high, 13 samples (37.1%) were stable, and MSI testing was not performed in 21 samples (60.0%). Table-II reports pathogenic/likely pathogenic variants and co-occurring mutations. Double gene mutations, specifically *KRAS* and *TP53*, were observed in 13 samples (37.1%), and *PIK3CA* and *KRAS* co-mutations were found in 2 samples (5.71%). A triple gene mutation, involving *KRAS*, *TP53*, and *PIK3CA*, was detected in 1 CRC tumor sample (3%). Table-III shows a significant association between *KRAS* mutations (66.7%) and older age (age > 50 years) (p < 0.03).

**Table-I:** Demographic characteristics and clinicopathological variables of Pakistani patients with late-stage colorectal cancer (n-35).

Characteristics	n	%
*Age(years)*		
<50	11	31.4
> 50	24	68.6
*Gender*		
Female	14	40.0
Male	21	60.0
*Histology*		
Adenocarcinoma	31	88.6
Mucinous adenocarcinoma	4	11.4
*AJCC stage CRC*		
Stage-III	2	9.5
Stage-IV	19	90.5
*CRC site*		
Proximal colon	5	21.7
Distal colon	14	60.9
Rectum	4	17.4
*Grade*		
Grade-1	5	21.7
Grade-2	14	60.9
Grade-3	4	17.4
*KRAS mutation*		
Mutation detected	19	54.3
Wild type	16	45.7
*TP53*		
High expression	23	65.7
Normal	12	34.3
*PIK3CA*		
Mutation detected	3	8.6
Wild type	32	91.4
*EGFR*		
Mutation detected	3	8.6
Wild type	32	91.4
*cKIT*		
Mutation detected	0	0.0%
Wild type	35	100
*NRAS*		
Mutation detected	3	8.6
Wild type	32	91.4
*BRAF*		
Mutation detected	0	0.0%
Wild type	35	100.0
*ERBB2*		
Mutation detected	0	0.0%
Wild type	35	100.0
*MET*		
Mutation detected	1	2.9
Wild type	34	97.1
*MSI*		
MSI-HIGH	1	2.9
STABLE	13	37.1
Not done	21	60.0

*Abbreviation:* **AJCC**: American Joint Committee on Cancer, **CRC**: Colorectal Cancer, **MSI**: Microsatellite instability.

**Table II:** Pathogenic and likely pathogenic variants identified in genes in Pakistani population with late-stage colorectal cancer (n=35)

	Gender	Age	Type	Gene	Codon Change	Protein Change	Transcript ID	MSI
1	Male	84	Adenocarcinoma	KRAS	c.35G>A	p.G12D(Gly12Asp)	NM_004985.5	N/A
				TP53	c.733G>A	p.G245S	NM_000546.6	
2	Male	59	Adenocarcinoma	KRAS	c.35G>C	p.G12A(Gly12Ala)	NM_004985.5	N/A
				TP53	c.638G>T	p.R213L	NM_000546.6	
3	Male	61	Adenocarcinoma	KRAS	c.35G>A	p.G12D(Gly12Asp)	NM_004985.5	Stable
				TP53	c.524G>A	p.R175H	NM_000546.6	
4	Male	32	Adenocarcinoma	TP53	c.824G>T	p.C275F	NM_000546.6	Stable
5	Male	63	Mucinous Adenocarcinoma	KRAS	c.35G>C	p.G12A	NM_004985.5	MSI-High
				TP53	c.1024C>T	p.R342	NM_000546.6	
6	Female	67	Adenocarcinoma	KRAS	c.34G>T	p.G12C	NM_004985.5	Stable
				TP53	c.659A>G	p.Y220C	NM_000546.6	
7	Female	75	Adenocarcinoma	TP53	c.818G >A	p.R273H	NM_000546.6	Stable
8	Female	44	Adenocarcinoma	TP53	c.483_489delCATC	p.Ile162SerfsTer6	NM_000546.6	Stable
9	Male	50	Adenocarcinoma	KRAS	c.34G>T	p.G12C	NM_004985.5	N/A
10	Female	48	Adenocarcinoma	KRAS	c.436G>A	p.A146T (Ala146Thr)	NM_033360.4	N/A
				TP53	c.586C>T	p.R196	NM_000546.6	
11	Male	63	Adenocarcinoma	TP53	c.711G>A	p.M227I (Met237Ile)	NM_000546.6	N/A
12	Female	63	Adenocarcinoma	KRAS	c.35G>T	p.G12V	NM_004985.5	N/A
13	Female	34	Adenocarcinoma	TP53	c.455C>T	p.P152L	NM_000546.6	N/A
14	Male	60	Adenocarcinoma	TP53	c.370delT	p.C124Afs*46	NM_001126112.2	N/A
15	Male	20	Adenocarcinoma	TP53	c.476C>T	p.A159V	NM_001126112.2	N/A
16	Male	76	Adenocarcinoma	KRAS	c.35G>A	p.G12D	NM_004985.5	N/A
				PIK3CA	c.1633G>A	p.E545K	NM_006218.4	
				TP53	c.733G>A	p.G245S	NM_000546.6	
17	Female	65	Adenocarcinoma	KRAS	c.183A>C	p.Q61H	NM_004985.5	Stable
				TP53	c.524G>A	p.R175H	NM_000546.6	
18	Male	65	Adenocarcinoma	KRAS	c.83G>A	p.G13D	NM_004985.5	N/A
				TP53	c.742C>T	p.R248W	NM_000546.6	N/A
19	Female	79	Adenocarcinoma	TP53	c.376T>G	p.Tyr126Asp	NM_000546.6	Stable
20	Male	53	Adenocarcinoma	KRAS	c.34G>T	p.Gly12Cys	NM_004985.5	N/A
				TP53	c.524G>A	p.Arg175His	NM_004985.5	N/A
21	Male	63	Adenocarcinoma	KRAS	c.35G>T	p.Gly12Val	NM_004985.5	Stable
				TP53	c.455dupC	p.Pro153Alafs*28	NM_000546.6	
22	Male	65	Adenocarcinoma	TP53	c.916C>T	p.Arg306	NM_000546.6	Stable
23	Male	59	Adenocarcinoma	NRAS	c.35G>A	p.Gly12Asp	NM_002524.5	N/A
24	Female	33	Adenocarcinoma	TP53	c.404G>T	p.Cys135Phe	NM_000546.6	N/A
25	Female	34	Adenocarcinoma	TP53	c.743G>A	p.Arg248Gln	NM_000546.6	N/A
26	Male	72	Adenocarcinoma	TP53	c.652G>A	p.v218m	NM_000546.6	N/A
27	Male	64	Mucinous Adenocarcinoma	KRAS	c.35G>A	p.G12D	NM_004985.5	N/A
28	Female	56	Adenocarcinoma	TP53	c.856G>A	p.Glu286Lys	NM_000546.6	N/A
29	Female	65	Adenocarcinoma	TP53	c.434T>C	p.Leu145Pro	NM_000546.6	N/A
				KRAS	c.437C>T	p.Ala146Val	NM_004985.5	
30	Male	69	Adenocarcinoma	TP53	c.844C>T	p.Arg282Trp	NM_000546.6	N/A
				KRAS	c.35G>T	p.Gly12Val	NM_004985.5	
31	Male	72	Adenocarcinoma	PIK3CA	c.1633G>A	p.Glu545Lys	NM_006218.4
				KRAS	c.35G>T	p.Gly12Val	NM_004985.5
32	Female	44	Mucinous Adenocarcinoma	TP53	c.586C>T	p.Arg196Ter	NM_000546.6	Stable
33	Male	48	Adenocarcinoma	PIK3CA	c.1633G>A	p.Glu545Lys	NM_006218.4	N/A
34	Male	27	Adenocarcinoma	WT	N/A	N/A	N/A	N/A
35	Male	54	Mucinous Adenocarcinoma	WT	N/A	N/A	N/A	N/A

*Abbreviation:* **WT**: wild type.

**Table III:** Association of age with gene mutations (KRAS &TP53) in CRC samples in Karachi Pakistan(n=35)

Gene Mutation	Category	Age <50 years	Age >50 years	p-value*
KRAS		n (%)	n (%)	0.03
	Yes	3(27.3)	16(66.7)	
	No	8(72.7)	8(33.3)	
TP53				0.08
	Yes	5(45.5)	18(75.0)	
	No	6(54.5)	6(25.0)	

* Chi square test.

## DISCUSSION

This study provides novel insights into the genomic landscape of late-stage CRC in a Pakistani cohort, a population previously underrepresented in such analyses. The high prevalence of *TP53* (65.7%) and *KRAS* (54.3%) mutations, along with the significant occurrence of double *KRAS/TP53* mutations (37.1%), highlights the aggressive nature of CRC in this population. The observed *KRAS* mutation are similar to Middle Eastern and South Asian populations, suggesting potential population-specific genetic predispositions.^8^,^9^ However, it exceeds that reported in Central Europe (25%) compared to the established range of at least 40-50% in the Western population like the USA.^10^ Conversely, the incidence of *TP53* inactivating mutations remained consistent across all populations (58%). A study in Tunisian population reported that *KRAS* somatic mutation was reported in the CRC tumor in 31.5 % (16/51) of the samples.^11^ In a study in the Chinese population, the mutation rates of *KRAS*, *NRAS*, and *BRAF* were 48.9%, 2.2%, and 3.2%, respectively, and the microsatellite instability-high rate was 9.5%.^12^ In another study in China, 38.6% of the CRC cases had *KRAS* mutations.^13^ Like our study, an analysis of the 99 Arab CRC cases revealed the most common prevalence of *KRAS* mutations (44.4%), followed by *TP53* mutations (52.5%) and lesser mutations in NRAS, and *BRAF* (4% each).^11^ Our data revealed higher *KRAS* and *TP53* mutation rates in the Pakistani population compared to Western populations, while *PIK3CA* and EGFR mutation rates were comparable. These variations can be attributed to genetic, environmental, and methodological differences, including sample size, late-stage CRC focus, and limited MSI testing.

In this study, the simultaneous presence of *KRAS* and *TP53* mutations was observed in 13% of samples. In contrast, in Tunisian population, simultaneous presence of *KRAS* and *TP53* mutations were detected in only 4% of tumor.^14^ Colorectal cancer patients harboring both *KRAS* mutations and high *TP53* expression exhibit a significantly poorer prognosis.^13^

Furthermore, a notable finding was the limited analysis of microsatellite instability (MSI) status, with only 2.9% classified as MSI-high and 37.1% identified as stable. MSI testing was not performed in over 60.0% of samples. Since MSI status can influence treatment options and prognosis, this highlights the need for a more comprehensive approach to genetic testing in Pakistani CRC patients.

CRC carcinogenesis involves complex interactions of oncogenes and tumor suppressor genes. Malignant transformation typically requires 4-5 cumulative gene mutations, with the total mutational burden impacting tumor behavior more than mutation order. According to the adenoma-carcinoma sequence (ACS) theory, adenoma precedes carcinoma, initiated by APC mutations in normal mucosa, followed by *KRAS* mutations in early to intermediate adenomas.^15^,^16^ CRC mutations in the *KRAS* gene are associated with older age group, and advanced cancer stage.^17^-^21^

### Limitations

There are important limitations of the study like the small size of the current study. This study utilized a small existing dataset comprising of 35 patients. Sample size calculation was not feasible due to several factors. Firstly, the study relied on an existing dataset with a limited number of patients meeting the inclusion criteria. Secondly, the high cost and limited accessibility of Next-Generation Sequencing (NGS) testing in Karachi, Pakistan. However, given the constraints of available data and the financial challenges associated with NGS testing, we believe that the analysis of this existing dataset still provides valuable insights about pathogenic mutations in Pakistani population with late-stage CRC. Another limitation was the limited analysis of MSI status which has a potential role in guiding treatment decisions, particularly in the era of immunotherapy.

### Strengths:

High quality of NGS testing done in the CAP accredited lab of AKUH and also the fact that it is first study that reports genomic landscape of CRC in the Pakistani population with its unique characteristics and assess simultaneous mutations of *TP53, PIK3CA, KRAS, and KRAS*.

This study’s findings have significant clinical implications for CRC management in Pakistan. High *KRAS* and *TP53* mutation rates suggest opportunities for targeted therapies, though *KRAS* mutations may confer resistance to EGFR-targeted treatments. The low MSI-high rate (2.9%) indicates a gap in molecular profiling, hindering the use of effective immunotherapies.

### Recommendations

Large scale studies are needed to define the Pakistani CRC genomic landscape and validate our findings. Further research should explore the therapeutic and prognostic roles of identified mutations. Increased MSI testing is crucial for comprehensive molecular profiling of Pakistani CRC patients.

## CONCLUSION

This study provides important insights into the characteristics and genetic profile of Pakistani CRC patients with late-stage disease, contributing to a better understanding of the disease in this population. The high prevalence of *TP53* and *KRAS* mutations underscores the importance of integration of both NGS and MSI testing into clinical practice to guide targeted therapies, personalize treatment strategies, and ultimately improve CRC patient outcomes in Karachi, Pakistan.

### Authors’ Contributions:

**ZA, AN and US** conceptualized the study, participated in data curation, data cleaning and data analysis, interpretation of data and manuscript writing. They are also responsible and accountable for the accuracy or integrity of the work.

**TM** participated in drafting and review of the manuscript.

All authors read and approved the final manuscript.

### List of Abbreviations:

**CRC:** Colorectal cancer,

**NGS:** Next generation sequencing,

**ACMG:** American College of Medical Genetics and Genomics,

**MSI:** Micro satellite Instability,

***TP53*:** Tumor protein 53, *KRAS* - *KRAS* protooncogene, GTPase

**NRAS:** NRAS protooncogene, EGFR - Epidermal growth factor receptor,

**GNA11:** Guanine nucleotide-binding protein, alpha-11,

**MET:** MET protooncogene, receptor tyrosine kinase,

***PIK3CA*:** Phosphatidylinositol 3-kinase, catalytic, alpha, RET - RET protooncogene, APC - APC regulator of wnt signaling pathway,

***BRAF*:** B-raf protooncogene, serine/threonine kinase,

**ERBB2:** Erb-b2 receptor tyrosine kinase 2,

**FOXL2:** forkhead transcription factor foxl2,

**GNAQ:** Guanine nucleotide-binding protein, q polypeptide, KIT - KIT protooncogene, receptor tyrosine kinase,

**PDGR1:** Platelet-derived growth factor receptor, alpha,

**CIN:** Chromosomal instability, **AKUH:** Aga Khan University hospital, **FFPE:** Formalin fixed paraffin embedded, **TST15:** TruSight Tumour 15 assay.
